# The vitality infant algorithm: A validated tool to classify food allergy outcomes in research studies

**DOI:** 10.1111/pai.70528

**Published:** 2026-10-23

**Authors:** Alexsandria Odoi, Jennifer J. Koplin, Victoria X. Soriano, Kayla M. Parker, Katrina J. Allen, Shyamali C. Dharmage, Katherine J. Lee, Jana K. Eckert, Audrey M. Walsh, Angela Young, Timothy W. Brettig, Rachel L. Peters, Kirsten P. Perrett

**Affiliations:** ^1^ Department of Paediatrics University of Melbourne Parkville Victoria Australia; ^2^ Population Allergy Group Murdoch Children's Research Institute Parkville Victoria Australia; ^3^ Centre for Food and Allergy Research (CFAR) Parkville Victoria Australia; ^4^ National Allergy Centre of Excellence (NACE) Parkville Parkville Victoria Australia; ^5^ Child Health Research Centre University of Queensland Brisbane Queensland Australia; ^6^ National Perinatal Epidemiology Unit, Nuffield Department of Population Health University of Oxford Oxford UK; ^7^ School of Population and Global Health University of Melbourne Parkville Victoria Australia; ^8^ Clinical Epidemiology & Biostatistics (CEBU) Murdoch Children's Research Institute Parkville Victoria Australia; ^9^ Department of Allergy and Immunology Royal Children's Hospital Parkville Victoria Australia

**Keywords:** algorithm, diagnostic, food allergy, infant, pediatric

## Abstract

**Background:**

Food allergy algorithms provide a systematic approach to assigning food allergy outcomes in research studies when oral food challenges (OFC) are not performed. Currently no such algorithms validated for infants exist. We aimed to develop and validate a research algorithm for classifying infant food allergies.

**Methods:**

An algorithm incorporating skin prick test (SPT), allergen ingestion, and reaction data was developed and validated by comparing peanut, cashew, egg, and cow's milk algorithm outcomes to OFC outcomes in infants from the Vitality (*n* = 285) (NCT02112734), EarlyNuts (*n* = 111) (ACTRN12618001990213), and HealthNuts (*n* = 191) studies.

**Results:**

Validation included 740 OFCs from 587 infants (191 SPT‐negative [0 mm] and 396 SPT‐positive [≥1 mm] infants; first‐OFC median age: 14.4 months). The algorithm assigned all SPT‐negative infants as tolerant, achieving 99.5% agreement with OFCs (peanut 100% [*n* = 135/135]; egg 98.2% [*n* = 55/56]). The algorithm assigned an allergic or tolerant outcome to 65% (93/144) of peanut SPT‐positive, 38% (41/108) cashew SPT‐positive, 81% (*n* = 209/258) egg SPT‐positive, and 90% (*n* = 35/39) milk SPT‐positive infants. Agreement with OFC was 81% (*n* = 75/93) for peanut, 88% (*n* = 36/41) for cashew, 88% (*n* = 184/209) for egg, and 97% (*n* = 34/35) for milk. Accuracy and areas under the curve (AUCs) were fair‐to‐excellent: peanut (AUC = 0.79 [95% confidence interval [CI]: 0.71–0.88]; sensitivity = 87% [95% CI: 75–95]; specificity = 72% [95% CI: 55–85]), cashew (AUC = 0.88 [95% CI: 0.77–0.98]; sensitivity = 84% [95% CI: 60–97]; specificity = 91% [95% CI: 71–99]); egg (AUC = 0.88 [95% CI: 0.81–0.94]; sensitivity = 88% [95% CI: 83–93]; specificity = 87% [95% CI: 70–96]); milk (AUC = 0.98 [95% CI: 0.94–1]; sensitivity = 96% [95% CI: 80–100]; specificity = 100% [95% CI: 69–100]).

**Conclusion:**

Using routinely collected data, this validated infant‐focused food allergy algorithm performed well in classifying peanut, cashew, egg, and cow's milk allergies, offering applicability in research settings.

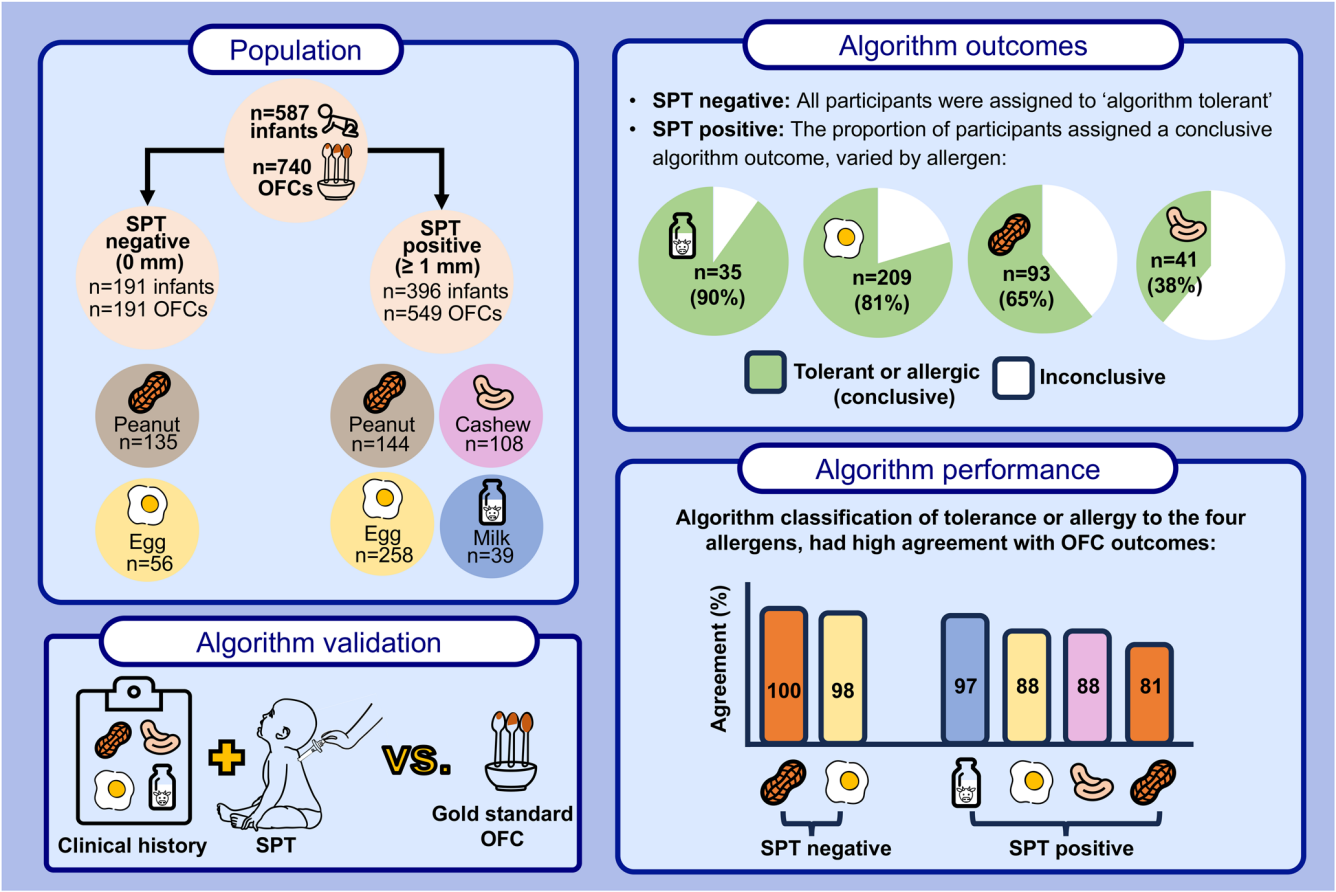

## Graphical abstract

This is the first algorithm validated for classifying food allergy in infants. Algorithm outcomes, derived from routine clinical data of SPT and clinical history, were validated against 740 OFCs from 587 infants. Overall, 69% of infants with a positive SPT (≥1 mm) received a conclusive algorithm outcome of allergic or tolerant for any allergen. Allergen‐specific conclusive outcomes ranged between 38% and 90%. The algorithm demonstrates good discriminative ability for infant food allergy classification, with consistently high agreement with OFC outcomes for cow's milk (97%), egg (88%), cashew (88%), and peanut (81%) allergy. OFC, Oral Food Challenge; SPT, Skin Prick Test.

**Abbreviations**
ASCIA: Australasian Society of Clinical Immunology and Allergy
AUC: Area under the Curve
BEEP: Barrier Enhancement for Eczema Prevention (study)
CI: Confidence Interval
EAT: Enquiring about Tolerance (study)
ED50: Eliciting Dose 50
IQR: Interquartile Range
IU: International Unit
LEAP: Learning Early About Peanut allergy
LTFU: Lost to follow‐up
MCRI: Murdoch Children's Research Institute
NPV: Negative Predictive Value
OFC: Oral Food Challenge
PPV: Positive Predictive Value
RCH: The Royal Children's Hospital
RCT: Randomized Controlled Trial
sIgE: Specific Immunoglobin E
SMS: Short Message Service
SPT: Skin Prick Test

**Key message**

Accurate food allergy diagnoses are crucial. While oral food challenges (OFCs) provide diagnostic certainty, they are not always performed in research. In the absence of OFC, reliable algorithms are needed to classify food allergy outcomes. We have validated an infant‐focused food allergy algorithm which incorporates routine data, including SPT and clinical history. Validated with 740 infant OFCs, the research algorithm demonstrates good discriminative ability for infant food allergy classification. The algorithm offers a resource‐efficient, validated framework for accurately classifying infant food allergy in research settings, when gold‐standard OFC are not performed.

## 1 INTRODUCTION

Food allergy is a significant health issue, affecting up to 10% of infants aged 12 months.^1^ In clinical and research settings, precise food allergy diagnosis is required. Whilst oral food challenges (OFC) are the gold standard for diagnosis of food allergy, they are also labour, time, and cost intensive and carry a risk of serious allergic reactions. As such, in a research setting, resource constraints^2^, ^3^ and participant/family preference mean this method is not always performed. When OFC are not completed, researchers can utilize a combination of routinely available clinical assessment methods, including caregiver report of allergic reactions and tests of sensitisation such as skin prick test (SPT) and blood tests for allergen specific IgE (sIgE), to guide food allergy diagnosis. However, as such approaches are not always standardized between different settings, algorithms implementing a systematic approach to food allergy diagnosis are needed.

Several diagnostic food allergy algorithms have been developed and validated for use, using data from both pediatric clinical and research study populations^4^, ^5^, ^6^, ^7^, ^8^, ^9^, ^10^ to predict food allergy where OFC is not performed. However, some rely on assessment techniques or data not readily available to all clinical or research settings,^4^, ^5^, ^6^, ^7^, ^8^, ^10^ are onerous,^10^ or can yield high rates of inconclusive outcomes.^10^ Importantly, none have been developed and validated specifically for use in infants.

The Vitality randomized controlled trial (RCT)^11^ was designed to determine whether daily supplementation of Vitamin D in the first year of life reduces the prevalence of food allergy at age 1 year. Open OFCs were used in conjunction with other diagnostic measures to define participant food allergy status. Loss to follow‐up (LTFU), partial participation, and impacts of stringent COVID‐19 restrictions prevented some infants from undergoing OFCs. Due to a lack of validated diagnostic food allergy algorithms for infants, it became imperative that we developed an a priori systematic approach, based on routinely collected allergy data, to aid infant food allergy classification for study participants who did not complete follow‐up or OFC. In this paper we:

1. Present the complete Vitality algorithm; a methodological approach for appointing the study primary outcome result of food allergy to all infants participating in the Vitality randomized controlled trial (RCT);
2. Validate the algorithm by assessing the agreement between the Vitality algorithm derived outcomes and gold standard OFC outcomes for 1‐year‐old research participants who underwent SPT.

## 2 METHODS

A previous version of this manuscript was posted as a preprint on Authorea.^12^ The present article has since undergone peer review and differs from the preprint in updated analysis and figures.

### 2.1 Algorithm overview

Our algorithm was developed by a panel consisting of pediatric allergists, allergy epidemiologists, data managers and clinical trial coordinators. The panel studied previous literature, including a comprehensive review of data collected in food allergy studies,^11^, ^13^ allergy profiles reported in local clinical^14^ and research cohorts,^1^, ^15^, ^16^ published pediatric food allergy algorithms,^6^, ^7^, ^8^, ^10^ models,^4^, ^5^, ^8^ scoring questionnaires,^9^ and population cohort algorithms.^1^, ^13^, ^17^ Using previously established allergy classification practices^1^, ^17^ as guiding frameworks, the panel collated the literature and used an iterative, consensus building approach to refine the algorithm. This included an adaptation and expansion of the original 2010 HealthNuts criteria^1^ which were developed for a single‐point questionnaire, during an era of low infant allergen ingestion,^18^ and at a time where published definitions of ingestion for tolerance were not reported. Key steps in our iterative process included replacing single sensitisation thresholds with allergen‐ and age‐specific values derived from literature and expert opinion. We consulted with allergists and a dietician to align questionnaire‐reported ingestion quantities, over time, with standardized allergen protein content and known eliciting doses (i.e., ED50), ensuring the algorithm reflected current patterns of allergen exposure.^18^

#### 2.1.1 Algorithm data and outcomes

The panel defined four key algorithm data inputs: caregiver report of tolerance, caregiver report of allergic reaction or anaphylaxis, participant SPT and participant OFC outcomes. Thresholds for these inputs, such as the minimum ingestion quantity^19^ and frequency for allergen tolerance, are described in Table 1, Tables S1 and S2. Five algorithm outcomes were defined as definite food allergy, probable food allergy, definite tolerant, probable tolerant and inconclusive food allergy (Table S3).

**TABLE 1:** Comparison of EarlyNuts and Vitality participant questionnaire ingestion quantities.

Allergen and form	Amount ingested (questionnaire responses)		Approximate measurement	Estimated protein (mg) content	Threshold met?	
	EarlyNuts	Vitality			ED50 discrete and cumulative^a^	Algorithm minimum for tolerance^b^
Peanut butter or Foods containing cashew	Bite or taste	Lick or taste	0.2 g	50 mg	‐	‐
	Small amount	1 bite	0.5 tsp.; 2.5 g	300 mg	Yes	‐
	Large amount	2 or more bites	1 tsp.+; 5 g+	600 mg+	Yes	Yes
Peanut nut or Cashew nut	‐	1 nut	1 g	250 mg	Yes	‐
	‐	2–4 nuts	2–4 g	500–1000 mg	Yes	‐
	‐	5 or more nuts	5 g	1200 mg+	Yes	Yes
Cow's milk	Sip	Sip	2.5 mL; 1/2 tsp	85 mg	‐	Yes
	1/4 cup	1/4 cup	125 mL	4250 mg	Yes	Yes
	At least 1/2 cup	At least 1/2 cup	250 mL+	8500 mg+	Yes	Yes
Dairy products^c^	Bite or taste	Lick or taste	1/2 tsp.; 2.5 mL	150 mg	Yes	‐
	Small amount	1 bite	1 tsp.; 5 ml	300 mg	Yes	‐
	Large amount	2 or more bites	2 tsp., 10 mL	600 mg+	Yes	Yes
Soft‐cooked egg	1 tsp	1 bite	5 g egg	600 mg	Yes	‐
	½ egg	½ egg	30 g egg	3800 mg	Yes	Yes
	Whole egg	Whole egg	60 g egg	7600 mg	Yes	Yes
‘Non‐cooked’ egg	Bite or taste	Lick or taste	1/2 tsp.; 2.5 g	300 mg	Yes	‐
	Small amount	1 bite	1 tsp.; 5 g	600 mg	Yes	‐
	Large amount	2 or more bites	2 tsp.; 10 g	1200 mg+	Yes	Yes

*Note*: Foods containing cashew includes cashew butter, mixed nut butters. Dairy products include yogurt, cheese, cream; Soft‐cooked egg includes soft boiled, poached, scrambled; non‐cooked egg includes Meringue, Pavlova or Macaroons. All estimated protein content for derived from Food Standards Australia New Zealand.

Abbreviations: g, grams; mg, milligrams; tsp, teaspoon.

^*a*^ Eliciting dose 50 (ED50.0) discrete/cumulative (2): peanut 165 mg/236 mg; cashew 139 mg/232 mg; egg 94.5 mg/134 mg; milk 125 mg/192 mg.

^*b*^ Amounts ingested must be consumed on at least 2 or more occasions, to meet the threshold for tolerance.

^c^ Estimated dairy protein content based on ‘natural yogurt, regular fat (3%), as this is a popular form of dairy product in infancy.

Participants with incongruous data, such as a report of allergen tolerance following reaction, or an inconclusive algorithm outcome with caregiver‐reported allergist diagnosis, underwent expert panel review (internal study allergist [K.P.P.] and external allergist [T.W.B]), to review participant data alongside additional clinical information if available, and appoint participants to one of the five algorithm outputs.

A hypothetical alternative algorithm has been proposed (Figure S1) for settings where serum sIgE is utilized. This alternative algorithm has not undergone validation as uptake of optional blood sample collection in the Vitality and EarlyNuts studies was limited (<30% of infants).

### 2.2 Study populations for algorithm validation

#### 2.2.1 The HealthNuts study

The HealthNuts study is a population‐based cohort study which recruited 5276, 11‐ to 15‐month‐old infants between August 2007 and September 2011.^20^ The study aimed to assess food allergy prevalence and infant allergen introduction and recruited infants attending their 12‐month immunisations at community‐run immunization sessions (Melbourne, Australia). Infants underwent a community SPT (peanut, egg, sesame, cow's milk and/or shellfish) after the parent/guardian provided written informed consent. Those with a SPT wheal ≥1 mm were invited to undergo a repeat clinic SPT and OFC at the Royal Children's Hospital (RCH). A random sample of infants with 0 mm community SPT at recruitment were also invited to the RCH to undergo peanut and raw egg OFCs to test the assumption that infants with 0 mm SPT were truly food tolerant.

#### 2.2.2 The EarlyNuts study

The EarlyNuts study is a population‐based cohort study of 11–15‐month‐olds (*n* = 1933) recruited between November 2016 and December 2019^13^ (ACTRN12618001990213). The EarlyNuts study shares a similar study design and recruitment framework to the HealthNuts study and aims to measure infant food allergy prevalence and allergen introduction, following changes to national infant feeding guidelines. Like the HealthNuts study, infants were recruited at community‐run immunization sessions (Melbourne, Australia) and underwent a community screening SPT (peanut, egg, cashew, cow's milk). Participants with a positive community SPT wheal (> = 1 mm) were invited to undergo a repeat SPT and OFC at the RCH.

#### 2.2.3 The Vitality trial

The Vitality trial (NCT02112734) is a double‐blind placebo‐controlled RCT^11^ which aims to determine if daily supplementation of 400 IU of vitamin D in the first year of life reduces the prevalence of food allergy at 12 months of age. Recruitment of 2739 infants from Melbourne (Australia) occurred between November 2014 and April 2022, with final follow up completed in August 2023. Written informed consent was obtained from the infant's parent/guardian, and randomization and study intervention commenced between 6 and 12 weeks of age. All infants were invited to a 12‐month clinic assessment at the RCH to undergo a clinic SPT, irrespective of their allergy or food ingestion history. OFC was offered to all infants with a positive clinic SPT (wheal > = 1 mm above the negative control). Algorithm development and validation was performed without knowledge of the Vitality trial treatment assignment, and prior to unblinding the trial data.

### 2.3 Allergy assessments and clinical history

Clinical history and allergy assessment data were captured from 6 to 23 months of age.

#### 2.3.1 Clinic skin prick test

Clinic SPT outcomes were included in algorithm validation. Clinic SPTs for all 3 studies were performed by research nurses, according to previously published protocols^15^ on the infant's upper back. Allergens tested varied by study; however, food allergens common to all included peanut, hen's egg, cashew, and cow's milk, plus a positive (histamine 10 mg/mL) and negative control (saline). SPTs were interpreted after 15 min. An SPT wheal > = 1 mm above the negative control was considered positive.^16^ Algorithm SPT thresholds are defined in Table S1.

#### 2.3.2 Oral food challenges

OFCs for all three studies were performed at our allergy research clinic at the RCH. OFCs were performed and interpreted in accordance with previously published protocols^1^, ^13^ (Table S4). Participants that were SPT‐positive to multiple allergens underwent multiple allergen OFCs, where possible.

Due to safety protocols, SPT‐positive infants with a recent allergic reaction (within 1 month of SPT for egg or milk, or 2 months for peanut or cashew), or a history of anaphylaxis (per the Australasian Society of Clinical Immunology [ASCIA]^21^ definition of anaphylaxis including: circulatory or respiratory compromise including difficulty breathing, stridor, wheeze, persistent cough, pallor, floppiness, collapse) to the allergen under investigation did not undergo OFC. During the OFC, participants ingested pre‐specified quantities of the food under investigation in steadily increasing portions every 15–20 min. The OFC was stopped and considered positive if participants experienced at least one of the following symptoms within 2 h of ingestion: 3 or more concurrent noncontact urticaria persisting for at least 5 min, perioral or periorbital angioedema, vomiting (excluding gag reflex), or anaphylaxis.^21^ OFCs were considered negative if all doses were successfully consumed without reaction symptoms. OFCs were considered inconclusive if the infant refused to initiate or complete the challenge.

#### 2.3.3 Questionnaires

Vitality caregivers completed questionnaires when their infants were aged 3, 6, 9, and 12 months, and EarlyNuts and HealthNuts caregivers when their infants were 12 months. Questionnaires captured demographic data and information about common allergens, such as ingestion patterns (timing, frequency, quantity) (Figures S2 and S3) and reactions (timing and type of symptoms).

### 2.4 Approach to validation

Algorithm validation assessed the agreement between algorithm‐derived outcomes (of definite or probable, allergic or tolerant) and gold‐standard OFC outcomes (of positive or negative). Inconclusive OFCs were not included in the validation. We assessed the agreement between algorithm‐derived outcomes and OFC outcomes for two scenarios (marked by the red stars in Figure 1):

1. SPT‐negative (0 mm) [Pathway A, branch 1]
2. SPT‐positive (> = 1 mm), eligible OFC not done [Pathway B, branch 3]

**FIGURE 1:**
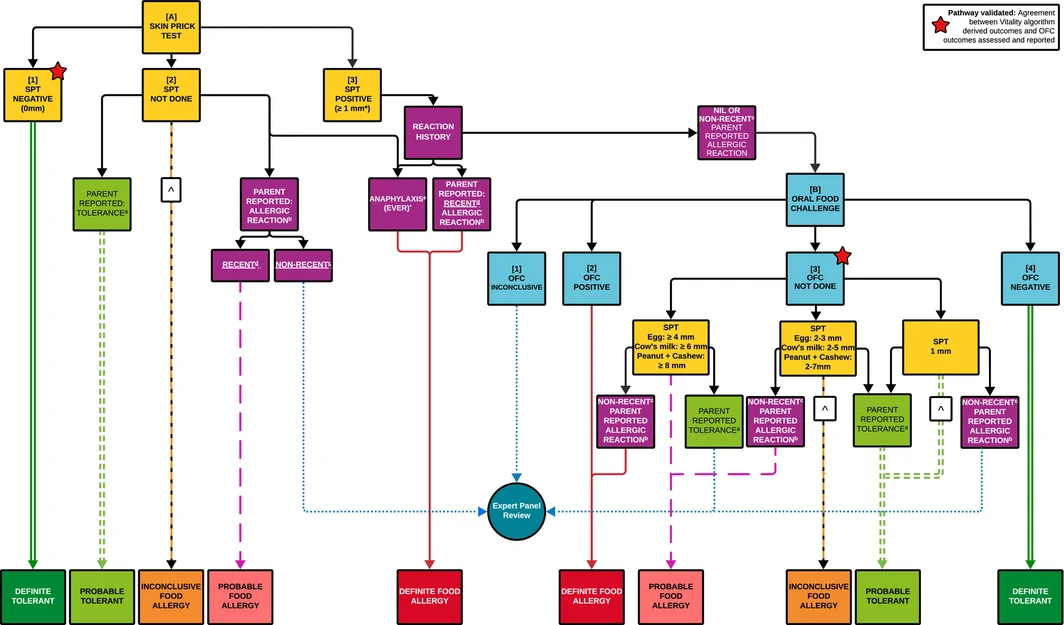
Algorithm for classifying infant food allergy. ^a^Parent reported tolerance: Two or more occasions where the following are ingested; 2 or more bites of peanut butter or foods containing cashew, or non‐cooked egg, or dairy containing foods (e.g. yogurt); or 5 or more peanuts or cashew nuts; or 1/2 a soft‐cooked egg; or 1‐sip of cow's milk. ^b^Parent reported allergic reaction: Caregiver‐report of symptoms consistent with sIgE allergic reaction of urticaria; perioral or periorbital angioedema or; vomiting, within 1 h of allergen ingestion. ^c^Non‐recent reaction: Reaction occurring more than 1 month (Egg and Cow's Milk) or more than 2 months (all other allergens) prior to the skin prick test (SPT). ^d^Recent reaction: Occurring within 1 month (Egg and Cow's Milk) or 2 months (all other allergens) of the skin prick test (SPT). ^e^Anaphylaxis: Defined as caregivers reporting symptoms consistent with the Australasian Society of Clinical Immunology (ASCIA) definition of anaphylaxis including circulatory or respiratory compromise. *Specific conditions apply for a report of anaphylaxis. Infants with a report of anaphylaxis and a SPT wheal size of 1 mm require expert panel review (not shown in figure). Infants with a report of anaphylaxis and a SPT wheal size of ≥2 mm, receive an algorithm classification of definite allergic (as per figure). ^No history of ingestion or tolerance, or history did not meet the algorithm‐specified threshold. mm, Millimeters; OFC, Oral food challenge; Image created in Lucid (lucid.co).

Scenario 1 assessed the accuracy of a 0 mm SPT in classifying tolerance, and scenario 2 assessed the accuracy of a positive SPT (> = 1 mm) combined with caregiver‐reported ingestion or reaction symptoms to classify tolerance or allergy outcomes.

#### 2.4.1 Statistical methods and analysis

OFC results remained blinded until algorithm outcomes were derived. Algorithm outcomes of definite tolerant and probable tolerant were aggregated into a single outcome (tolerant) for all analyses. Inconclusive algorithm outcomes were excluded from all analyses.

Algorithm‐derived outcomes for each food allergen were considered individually and then combined into an outcome of ‘any allergy’. For ‘any allergy’, infants were deemed allergic if they were allergic to at least one food and tolerant if they were tolerant to all foods tested.

The primary analysis combined definite and probable allergic algorithm outcomes into one single outcome of ‘allergic’. A sensitivity analysis was performed assessing the algorithm classification of definite allergic only (excluding probable allergic). Sensitivity, specificity, positive (PPV) and negative predictive values (NPV), and area under the curve (AUC) for predicting OFC outcomes were obtained for both the primary and sensitivity analysis using the ‘*diagt*’ package in Stata.^22^ Sensitivity and specificity were interpreted as: 90%–100% very high; >80%–90% high; moderate 65%–80%; low <65%.^23^ AUC were interpreted as follows: 1.0 perfect; 0.9–0.99 excellent; 0.8–0.89 good; 0.7–0.79 fair; 0.51–0.69 poor; and 0.5, test of no value.^24^ We performed a complete case analysis. All analyses were performed using Stata version 18.0 (StataCorp, College Station, TX).

### 2.5 Ethical approval

The RCH Human Research Ethics Committee (HREC) approved the Vitality trial on 9 September 2014 (#34168A) and EarlyNuts study on 9 September 2016 (#36160A). HREC approval for HealthNuts was obtained from the RCH HREC (ref. no. 27047A), the Office for Children, Government of Victoria HREC (ref. no. CDF/07/492), and the Department of Human Services, Government of Victoria (ref. no. 10/07). Parents/guardians from all three studies provided informed written consent for study participation and use of participant data that fell within the original study aims of assessing allergic diseases.

## 3 RESULTS

### 3.1 Participant demographics and OFC summary

Tables 2 and 3 summarize demographic and OFC characteristics of SPT‐negative (0 mm) HealthNuts participants, and the SPT‐positive (≥1 mm) EarlyNuts and Vitality participants included in algorithm validation. Altogether, 191 HealthNuts participants completed 191 peanut or raw egg OFCs, 111 EarlyNuts participants completed 166 peanut, cashew, raw egg or milk OFCs, and 285 Vitality participants completed 383 peanut, cashew, raw egg or milk OFCs (Table 3).

**TABLE 2:** Demographic and allergy history characteristics of 587 1‐year‐old infants from the HealthNuts, EarlyNuts study and Vitality trial who underwent peanut, cashew, egg and milk OFC.

Participant characteristic	HealthNuts (*n* = 191)	EarlyNuts (*n* = 111)	Vitality (*n* = 285)
	*n*/*N* (%)	*n*/*N* (%)	*n*/*N* (%)
Infant sex (male)	94/191 (49.5)	57/111 (51.4)	165/285 (57.9)
Infant has siblings	96/191 (50.3)	40/104 (38.5)	120/284 (42.3)
Family history of food allergy			
Immediate family member^a^	46/191 (24.1)	27/109 (24.8)^i^	103/278^i^ (37.1)
Sibling(s)	15/96 (15.6)	4/33 (10.3)^i^	43/120^i^ (35.8)
Parent	35/191 (18.3)	16/102 (15.7)^i^	71/246^i^ (28.9)
Parents country of birth			
Both Australia	136 (71.2)	42 (37.8)	156 (54.7)
Both East Asia, or one East Asia and one Australia^b^	8 (4.2)	35 (31.5)	36 (12.6)
One Australia, and one other^g^ or unknown^c^	35 (18.3)	17 (15.3)	61 (21.4)
One East Asia, and one other^g^ or unknown^d^	1 (0.5)	3 (2.7)	8 (2.8)
Both other, or one other^g^ and one unknown^e^	11 (5.8)	7 (5.3)	23 (8.1)
Both unknown^f^	‐	7 (6.3)	1 (0.4)
Socioeconomic status by postcode^h^			
Median (IQR)	9 (7–10)^j^	8 (7–9)	8 (7–10)^k^

*Note*: Total numbers may vary due to missing data.

Abbreviations: CI, Confidence interval; IQR, interquartile range; OFC, Oral Food Challenge; RCT, Randomized Control Trial.

^a^ Immediate family includes the infant's mother, father, and siblings.

^b^ Both East Asia, or one East Asia and one Australia refers to infants with both parents born in East Asia or, infants with 1 parent born in East Asia and their second parent born in Australia.

^c^ One Australia, and one other or unknown refers to infants with only 1 parent born in Australia, and second parent born elsewhere (but not in East Asia) or their country/region of birth is unknown.

^d^ One Australia, and one other or unknown refers to infants with only 1 parent born in East Asia, and the second parent born elsewhere (but not in Australia) or their country/region of birth is unknown.

^e^ Both other, or one other and one unknown refers to infants with one or both parents born in a region/country that does not include Australia and/or East Asia, and the parents' country of birth is unknown.

^f^ Both unknown refers to infants where both parents' country of birth is unknown.

^g^ ‘Other’ country/region of birth refers to all combinations of parents countries, excluding one or both parents born in Australia and/or East Asia. Other countries/regions include: Africa, Europe, South America, South Asia, Middle East, North America; Oceania; United Kingdom/Britain.

^h^ Australian Bureau of Statistics measure of socioeconomic status decile in the Index of Relative Socio‐economic Advantage and Disadvantage (IRSAD) statistical area 1 for 2006 and 2016, based on family postcodes, with scale from 1 reflecting most disadvantage to 10 least disadvantage.

^i^ Category totals and subcategory totals may vary as some caregivers reported immediate family history of food allergy, however, did not provide a response for which family member (parent or sibling) had the food allergy.

^j^ *n* = 190.

^k^ *n* = 284.

**TABLE 3:** Summary of the 740 oral food challenge results undertaken by 587 infants, by research study.

Participant characteristic	SPT negative	SPT positive	
	HealthNuts	EarlyNuts	Vitality
Oral food challenge, *n*			
Total participants assessed by OFC	191	111	285
Total OFCs performed	191	166	383
Positive by OFC, *n*/*N* (%)			
Peanut	0/135 (0)	29/48 (60)	50/96 (52)
Cashew	–^a^	14/36 (39)	29/72 (40)
Egg (raw)	1/56 (1.8)	62/72 (86)	152/186 (82)
Cow's milk (raw)	–^a^	5/10 (50)	24/29 (83)
All participants	1/191 (0.5)	80/111 (72)	199/283 (70)
All OFCS	1/191 (0.5)	110/166 (66)	255/383 (67)
Age at first OFC (months), median (IQR)			
All OFCs	14.4 (13.7, 15.2)^b^	14.1 (13.4, 15.1)	14.4 (13.4, 15.6)

Abbreviations: IQR, interquartile range; OFC, Oral food challenge.

^a^ Not performed in the study.

^b^ *n* = 189 due to missing data.

Median age of first OFC was similar in the three studies (14.4 [13.5–15.4] months) (Table 2). For the SPT‐negative HealthNuts participants, the proportion of negative OFCs was 100% for peanut (*n* = 135/135) and 99.5% for raw egg (*n* = 55/56) (Table 3). For the SPT‐positive participants, the proportion of positive OFCs was highest for raw egg (82.9%), followed by milk (74.4%), peanut (54.9%), and cashew (39.8%); with 69.7% of these participants deemed allergic by OFC to at least one allergen (Table 3).

### 3.2 Algorithm validation

#### 3.2.1 SPT negative: Peanut and egg validation

OFCs from HealthNuts participants were used to validate the assumption of allergen tolerance across 191 negative peanut or egg SPTs (represented by pathway “A: SPT, branch 1: SPT NEGATIVE”, Figure 1). Peanut 0 mm SPT demonstrated very high NPV in predicting negative OFC (100% [95% CI, 97.3–100]) (Table 4). Egg 0 mm SPT showed high NPV in predicting negative OFC (98.2% [95% CI, 90.4–100.0]) (Table 4), with one possible false negative out of 56 OFCs due to 1 infant report of a late reaction following egg OFC with SPT 0 mm.

**TABLE 4:** Algorithm validation outcome for SPT‐negative (0 mm) peanut, egg allergy and ‘any allergy’ participants from the HealthNuts study.

Allergen	Algorithm outcome	OFC outcome (*n*)		Total conclusive algorithm outcomes (*n*)	Agreement (95 CI)	Sensitivity (95 CI)	Specificity (95 CI)	PPV (95 CI)	NPV (95 CI)	AUC (95 CI)
		Positive	Negative							
Peanut	Allergic	0	0	0	100 (97.3–100)	*	100 (97.3–100)	*	100 (97.3–100)	*
	Tolerant	0	135	135						
	Total	0	135	135						
Egg	Allergic	0	0	0	98.2 (90.4–100)	*	100 (93.5–100)	*	98.2 (90.4–100)	*
	Tolerant	1	55	56						
	Total	1	55	56						
Any Allergy^a^	Allergic	0	0	0	99.5 (97.1–100)	*	100 (98.1–100)	*	99.5 (97.1–100)	*
	Tolerant	1	190	191						
	Total	1	190	191						

^a^ Any Allergy combines all participants peanut and egg allergy outcomes. Calculations of algorithm agreement, sensitivity, specificity, positive predictive values (PPV), negative predictive values (NPV), odds ratio (OR), and Area Under the Curve (AUC) include algorithm classifications of tolerant (and exclude inconclusive outcomes).

* Unable to calculate.

#### 3.2.2 SPT positive: Validation overview

Peanut, cashew, egg and milk OFCs (*n* = 549) from 396 EarlyNuts and Vitality participants (Tables 3 and 5) were used to validate the algorithm's ability to assign allergic or tolerant outcomes, to SPT‐positive infants (represented by pathway “B: ORAL FOOD CHALLENGE, branch 3: OFC NOT DONE”, Figure 1). The algorithm classified tolerance or allergy in 378/549 (68.9%) OFCs (Table 5). Of these, 329/378 (87.0% [95% CI 83.2–90.3]) algorithm outcomes agreed with the OFC outcome (Table 5). Seventeen of the 102 negative‐OFCs (16.7%) were misclassified as positive, due mostly to peanut misclassification (64.7%, *n* = 11) (Table 6). Thirty‐two of the 276 (11.6%) positive‐OFCs were algorithm misclassified as tolerant, largely driven by raw‐egg misclassification (65.6%, *n* = 21/32). These algorithm‐misclassified positive‐OFCs recorded mild reactions of urticaria (*n* = 23), peri‐oral or peri‐orbital angioedema (*n* = 6), transient cough (*n* = 1) or rhinorrhoea (*n* = 1), or delayed‐onset vomiting (2.5–4.0 h following OFC, *n* = 2).

**TABLE 5:** Algorithm derived outcomes compared to OFC confirmed allergy for peanut, cashew, egg, cow's milk and ‘any allergy’ SPT‐positive (≥1 mm) participants in the EarlyNuts and Vitality studies.

Allergen	Combined^a^ (*n* = 396)				EarlyNuts (*n* = 111)				Vitality (*n* = 285)			
	Algorithm outcome	OFC outcome *n* (%)		Total number of algorithm outcomes	Algorithm outcome	OFC outcome *n* (%)		Total number of algorithm outcomes	Algorithm outcome	OFC outcome *n* (%)		Total number of algorithm outcomes
		Positive (Allergic)	Negative (Tolerant)			Positive (Allergic)	Negative (Tolerant)			Positive (Allergic)	Negative (Tolerant)	
Peanut	Definite Allergic	8 (10.1)		8 (5.6)	Definite Allergic	5 (17.2)		5 (10.4)	Definite Allergic	3 (6.0)		3 (3.1)
	Probable Allergic	39 (49.4)	11 (16.9)	50 (34.7)	Probable Allergic	9 (31.0)	1 (5.3)	10 (20.8)	Probable Allergic	30 (60.0)	10 (21.7)	40 (41.7)
	Tolerant	7 (8.9)	28 (43.1)	35 (24.3)	Tolerant	3 (10.3)	8 (42.1)	11 (22.9)	Tolerant	4 (8.0)	20 (43.5)	24 (25.0)
	Inconclusive	25 (31.6)	26 (40.0)	51 (35.4)	Inconclusive	12 (41.4)	10 (52.6)	22 (45.8)	Inconclusive	13 (26.0)	16 (34.8)	29 (30.2)
	Total OFCs	79	65	144	Total OFCs	29	19	48	Total OFCs	50	46	96
Cashew	Definite Allergic	1 (2.3)		1 (0.9)	Definite Allergic				Definite Allergic	1 (3.4)		1 (1.4)
	Probable Allergic	15 (34.9)	2 (3.1)	17 (15.7)	Probable Allergic	4 (28.6)	1 (4.5)	5 (13.9)	Probable Allergic	11 (37.9)	1 (2.3)	12 (16.7)
	Tolerant	3 (7.0)	20 (30.8)	23 (21.3)	Tolerant		7 (31.8)	7 (19.4)	Tolerant	3 (10.3)	13 (30.2)	16 (22.2)
	Inconclusive	24 (58.8)	43 (66.2)	67 (62.0)	Inconclusive	10 (71.4)	14 (63.6)	24 (66.7)	Inconclusive	14 (48.3)	29 (67.4)	43 (59.7)
	Total OFCs	43	65	108	Total OFCs	14	22	36	Total OFCs	29	43	72
Egg	Definite Allergic	36 (16.8)	1 (2.3)	37 (14.3)	Definite Allergic	5 (8.1)		5 (6.9)	Definite Allergic	31 (20.4)	1 (2.9)	32 (17.2)
	Probable Allergic	121 (56.5)	3 (6.8)	124 (48.1)	Probable Allergic	29 (46.8)		29 (40.3)	Probable Allergic	92 (60.5)	3 (8.8)	95 (51.1)
	Tolerant	21 (9.8)	27 (61.4)	48 (18.6)	Tolerant	12 (19.4)	8 (80.0)	20 (27.8)	Tolerant	9 (5.9)	19 (55.9)	28 (15.1)
	Inconclusive	36 (16.8)	13 (29.5)	49 (19.0)	Inconclusive	16 (25.8)	2 (20.0)	18 (25.0)	Inconclusive	20 (13.2)	11 (32.4)	31 (16.7)
	Total OFCs	214	44	258	Total OFCs	62	10	72	Total OFCs	152	34	186
Cow's Milk	Definite Allergic	8 (27.6)		8 (20.5)	Definite Allergic	1 (20.0)		1 (10.0)	Definite Allergic	7 (29.2)		7 (24.1)
	Probable Allergic	16 (55.2)		16 (41.0)	Probable Allergic	3 (60.0)		3 (30.0)	Probable Allergic	13 (54.2)		13 (44.8)
	Tolerant	1 (3.4)	10 (100.0)	11 (28.2)	Tolerant		5 (100.0)	5 (50.0)	Tolerant	1 (4.2)	5 (100.0)	6 (20.7)
	Inconclusive	4 (13.8)		4 (10.3)	Inconclusive	1 (20.0)		1 (10.0)	Inconclusive	3 (12.5)		3 (10.3)
	Total OFCs	29	10	39	Total OFCs	5	5	10	Total OFCs	24	5	29
All OFCs^b^	Definite Allergic	53 (14.5)	1 (0.5)	54 (9.8)	Definite Allergic	11 (10.0)		11 (6.6)	Definite Allergic	42 (16.5)	1 (0.8)	43 (11.2)
	Probable Allergic	191 (52.3)	16 (8.7)	207 (37.7)	Probable Allergic	45 (40.9)	2 (3.6)	47 (28.3)	Probable Allergic	146 (57.3)	14 (10.9)	160 (41.8)
	Tolerant	32 (8.8)	85 (46.2)	117 (21.3)	Tolerant	15 (13.6)	28 (50.0)	43 (25.9)	Tolerant	17 (6.7)	57 (44.5)	74 (19.3)
	Inconclusive	89 (24.4)	82 (44.6)	171 (31.1)	Inconclusive	39 (35.5)	26 (46.4)	65 (39.2)	Inconclusive	50 (19.6)	56 (43.8)	106 (27.7)
	Total OFCs	365	184	549	Total OFCs	110	56	166	Total OFCs	255	128	383
Any Allergy^c^	Definite Allergic	51 (17.9)		51 (12.9)	Definite Allergic	10 (12.5)		10 (9.0)	Definite Allergic	41 (20.0)		41 (14.4)
	Probable Allergic	151 (53.0)	14 (12.6)	165 (41.7)	Probable Allergic	37 (46.2)	3 (9.7)	40 (36.0)	Probable Allergic	114 (55.6)	11 (13.8)	125 (43.9)
	Tolerant	22 (7.7)	50 (45.0)	72 (18.2)	Tolerant	11 (13.8)	14 (45.2)	25 (22.5)	Tolerant	11 (5.4)	36 (45.0)	47 (16.5)
	Inconclusive	61 (21.4)	47 (42.3)	108 (27.3)	Inconclusive	22 (27.5)	14 (45.2)	36 (32.4)	Inconclusive	39 (19.0)	33 (41.2)	72 (25.3)
	Total OFCs	285	111	396	Total OFCs	80	31	111	Total OFCs	205	80	285

^a^ Combined: All participant outcomes from the EarlyNuts and Vitality, grouped together.

^b^ All OFCS: Total OFCs and allergy outcomes across all allergens.

^c^ Any Allergy: Allergy status by participant, combining allergy outcome for participants with multiple oral food challenges (OFC): infants are deemed allergic if allergic to at least one food, and tolerant if tolerant to all foods.

**TABLE 6:** Algorithm validation outcomes for peanut, cashew, egg, cow's milk allergy and ‘any allergy’, in SPT‐positive (≥1 mm) participants from the EarlyNuts, and Vitality studies. Algorithm outcomes of definite and probable allergy are combined into ‘allergic’ (primary analysis).

Allergen	Study	Algorithm outcome	OFC Outcome (*n*)		Total conclusive algorithm outcomes (*n*)	Agreement (95 CI)	Sensitivity (95 CI)	Specificity (95 CI)	PPV (95 CI)	NPV (95 CI)	AUC (95 CI)
			Positive	Negative							
Peanut	EarlyNuts	Allergic	14	1	15	84.6 (65.1–95.6)	82.4 (56.6–96.2)	88.9 (51.8–99.7)	93.3 (68.1–99.8)	72.7 (39.0–94.0)	0.86 (0.71–1)
		Tolerant	3	8	11						
		Total	17	9	26						
	Vitality	Allergic	33	10	43	79.1 (67.4–88.1)	89.2 (74.6–97.0)	66.7 (47.2–82.7)	76.7 (61.4–88.2)	83.3 (62.6–95.3)	0.78 (0.68–0.88)
		Tolerant	4	20	24						
		Total	37	30	67						
	Combined^a^	Allergic	47	11	58	80.6 (71.1–88.1)	87.0 (75.1–94.6)	71.8 (55.1–85.0)	81.0 (68.6–90.1)	80.0 (63.1–91.6)	0.79 (0.71–0.88)
		Tolerant	7	28	35						
		Total	54	39	93						
Cashew	EarlyNuts	Allergic	4	1	5	91.7 (61.5–99.8)	100 (39.8–100)	87.5 (47.4–99.7)	80.0 (28.4–99.5)	100 (59.0–100)	0.94 (0.82–1)
		Tolerant	0	7	7						
		Total	4	8	12						
	Vitality	Allergic	12	1	13	86.2 (68.3–96.1)	80.0 (51.9–95.7)	92.9 (66.1–99.8)	92.3 (64.0–99.8)	81.3 (54.4–96.0)	0.86 (0.74–0.99)
		Tolerant	3	13	16						
		Total	15	14	29						
	Combined^a^	Allergic	16	2	18	87.8 (74.0–95.9)	84.2 (60.4–96.6)	90.9 (70.8–98.9)	88.9 (65.3–98.6)	87.0 (66.4–97.2)	0.88 (0.77–0.98)
		Tolerant	3	20	23						
		Total	19	22	41						
Egg	EarlyNuts	Allergic	34	0	34	77.8 (64.4–88.0)	73.9 (58.9–85.7)	100 (63.1–100)	100 (89.7–100)	40.0 (19.1–64.0)	0.87 (0.81–0.93)
		Tolerant	12	8	20						
		Total	46	8	54						
	Vitality	Allergic	123	4	127	91.6 (86.1–95.5)	93.2 (87.5–96.8)	82.6 (61.2–95.1)	96.9 (92.1–99.1)	67.9 (47.7–84.1)	0.88 (0.80–0.96)
		Tolerant	9	19	28						
		Total	132	23	155						
	Combined^a^	Allergic	157	4	161	88.0 (82.9–92.1)	88.2 (82.5–92.6)	87.1 (70.2–96.4)	97.5 (93.8–99.3)	56.3 (41.2–70.5)	0.88 (0.81–0.94)
		Tolerant	21	27	48						
		Total	178	31	209						
Cow's milk	EarlyNuts	Allergic	4	0	4	100 (63.4–100)	100 (39.8–100)	100 (47.8–100)	100 (39.8–100)	100 (47.8–100)	1 (1–1)
		Tolerant	0	5	5						
		Total	4	5	9						
	Vitality	Allergic	20	0	20	96.2 (80.3–99.9)	95.2 (76.2–99.9)	100 (47.8–100)	100 (83.2–100)	83.3 (35.9–99.6)	0.98 (0.93–1)
		Tolerant	1	5	6						
		Total	21	5	26						
	Combined^a^	Allergic	24	0	24	97.1 (85.1–99.9)	96.0 (79.7–99.9)	100 (69.2–100)	100 (85.8–100)	90.9 (58.7–99.8)	0.98 (0.94–1)
		Tolerant	1	10	11						
		Total	25	10	35						
Any Allergy^b^	EarlyNuts	Allergic	47	3	50	81.3 (70.7–89.4)	81.0 (69.6–90.1)	82.4 (56.6–96.2)	94.0 (83.5–98.8)	56.0 (34.9–75.6)	0.82 (0.71–0.92)
		Tolerant	11	14	25						
		Total	58	17	75						
	Vitality	Allergic	155	11	166	89.7 (84.8–93.4)	93.4 (88.5–96.7)	76.6 (62.0–87.7)	93.94 (88.5–96.7)	76.6 (62.0–87.7)	0.85 (0.79–0.91)
		Tolerant	11	36	47						
		Total	166	47	213						
	Combined^a^	Allergic	202	14	216	87.5 (83.1–91.1)	90.2 (85.5–93.7)	78.1 (66.0–87.5)	93.5 (89.4–96.4)	69.4 (57.5–79.8)	0.84 (0.79–0.90)
		Tolerant	22	50	72						
		Total	224	64	288						

*Note*: Calculations of algorithm agreement, sensitivity, specificity, positive predictive values (PPV), negative predictive values (NPV), odds ratio (OR) and Area Under the Curve (AUC) include algorithm classifications of allergic and tolerant. References FSANZ^19^ and Houben et al.^34^

^a^ All participant outcomes from the EarlyNuts and Vitality, grouped together.

^b^ Any Allergy combines allergy outcome for participants with multiple oral food challenges (OFC): infants are deemed allergic if allergic to at least one food, and tolerant if tolerant to all foods.

Most SPT wheals (58.3%) measured within the moderate range for their respective food allergen (> = 2 mm to <95% PPV), 35% were high (wheal > = 95% PPV), and 7% were low (1 mm) (Table S5). The algorithm derived a conclusive outcome for 46.9% of moderately sized SPTs (82.6% milk, 55.3% peanut, 51.5% egg, 20.2% cashew) at 79.3% overall agreement (100% milk, 76% peanut, 75% egg, and 82% cashew) (Table S5). Algorithm outcomes associated with moderate SPT results accounted for 36.3% of the algorithm agreement with OFC outcomes, whilst algorithm outcomes associated with high and low SPT results accounted for 54.3% and 9.4% of agreement respectively (Table S5).

Expert panel review outcomes were included in all analysis. Expert panel review was required for 52 of the 549 (9.5%) outcomes, largely due to high SPT (> = 95% PPV) with caregiver‐reported tolerance (Table S6). Five review panel outcomes were assigned as inconclusive, with 85.0% (*n* = 44/52) of the remaining review panel outcomes correctly identifying tolerance or an allergy compared to OFC outcome.

#### 3.2.3 SPT positive: Peanut allergy validation

The algorithm assigned outcomes of allergy or tolerance in 64.5% (*n* = 93/144) of peanut SPT‐positive infants (Table 5). It demonstrated high sensitivity (EarlyNuts 82.4% [95% CI, 56.6–96.2] *n* = 26; Vitality 89.2% [95% CI, 74.6–97.0] *n* = 67) and moderate to high specificity (EarlyNuts: 88.9% [95% CI, 51.8–99.7]; Vitality 66.7% [95% CI, 47.2–82.7]) (Table 6). The algorithm correctly classified 75 of 93 (accuracy 80.6% [95% CI, 71.1–88.1]) peanut outcomes across the EarlyNuts and Vitality groups with an AUC of 0.79 (95% CI, 0.71–0.88). Of the 11 OFC‐negative infants algorithm‐misclassified as peanut allergic, 10 experienced sub‐threshold symptoms at OFC (e.g. erythema or <3 transient hives). Ten OFC‐negative infants also had caregiver‐report of allergic reaction symptoms that met our threshold for IgE‐mediated allergy; 6 of whom received a prior doctor diagnosis, external to the study, of peanut allergy. Of the seven infants algorithm‐misclassified as peanut tolerant, six had caregiver‐report of sufficient peanut ingestion (Table S5): 4 reporting sub‐threshold reaction symptoms (rash) after ingestion, and 2 reporting no symptoms (data not shown). All other infants (*n* = 23) with sufficient peanut ingestion, were accurately algorithm classified as tolerant (Table S5).

#### 3.2.4 SPT positive: Cashew allergy validation

The algorithm assigned outcomes of allergy or tolerance in 38.0% (*n* = 41/108) of the cashew SPT‐positive infants (Table 5). Combined, the algorithm demonstrated high accuracy with 87.8% (95% CI, 74.0–95.9) agreement, 84.2% sensitivity (95% CI, 60.4–96.6), 90.9% specificity (95% CI, 70.8–98.9), 88.9% PPV (95% CI, 65.3–98.6), and 87.0% NPV (95% CI, 66.4–97.2), and good AUC of 0.88 (95% CI, 0.77–0.98) (Table 6). Of the 5 cashew algorithm misclassifications (1 from EarlyNuts and 4 from Vitality), 2 OFC‐negative infants were algorithm‐misclassified as allergic due to high sensitisation (SPT >= 8 mm) and no cashew ingestion ever (Table S5). Three OFC positive, moderately sensitized (2–7 mm) infants were algorithm‐misclassified as cashew tolerant due to parent reported ingestion history that met tolerance thresholds (Table S5). Conversely, all other infants with threshold‐sufficient cashew ingestion (*n* = 10) were accurately classified as tolerant (Table S5).

#### 3.2.5 SPT positive: Egg allergy validation

The algorithm assigned outcomes of allergy or tolerance to 81.0% (*n* = 209/258) of egg SPT‐positive infants (Table 5). It correctly classified 184 of 209 (88.0%) egg outcomes, resulting in high algorithm sensitivity (88.2% [95% CI, 82.5–92.6]) and specificity (87.1% [95% CI, 70.2–96.4]), very high PPV (97.5% [95% CI, 93.8–99.3]), low NPV (56.3% [95% CI, 41.2–70.5]), and good AUC (0.88 [95% CI, 0.81–0.94]) (Table 6). Eighteen low (1 mm, *n* = 6) to moderately (2–3 mm, *n* = 12) sensitized OFC‐positive infants were algorithm‐misclassified as egg tolerant; 13/18 met our egg ingestion threshold for tolerance, 2/18 demonstrated convincing recent tolerance (via ingestion history) after prior allergic reaction (per expert panel), and 3/18 were 1 mm‐sensitized with no or sub‐threshold history of tolerance or allergy (Table S5). Four OFC‐negative infants were algorithm‐misclassified as egg allergic, including one moderately sensitized infant with parent report of doctor diagnosis of egg allergy, and 3 highly sensitized (SPT > = 4 mm) infants (Table S5).

History of soft‐cooked egg ingestion was used to estimate raw‐egg OFC tolerance, with 54 (38%) egg‐sensitized participants meeting our egg‐tolerance threshold. Twenty‐one of these participants were highly sensitized (> = 4 mm), 26 moderately sensitized (2–3 mm), and seven were 1 mm egg sensitized (Table S5). The expert panel took a conservative approach to favor a raw‐egg allergic outcome for participants highly sensitized by egg SPT (> = 95% PPV) with soft‐cooked egg tolerance. This approach was favorable for most cases (16/21) (Table S5). For low‐to‐moderately sensitized participants, history of soft‐cooked egg tolerance helped to accurately classify 20 infants as tolerant but misclassified 13 (60.5% accuracy) (Table S5). As previously described, all 1 mm‐sensitized participants with *no* or sub‐threshold history of soft‐egg ingestion (or reaction) were classified as egg tolerant, however only half (3/6) agreed with actual OFC outcomes (i.e., three 1 mm infants were egg allergic by OFC) (Table S5).

#### 3.2.6 SPT positive: Cow's milk allergy validation

The algorithm assigned outcomes of allergy or tolerance to 89.7% (*n* = 35/39) of milk SPT‐positive infants (Table 5). The algorithm performed strongest for classifying cow's milk, compared to all other allergens, with very high agreement (97.1% [95% CI, 85.1–99.9]), sensitivity, specificity, PPV, and NPV, and an AUC of 0.98 (0.94–1) (Table 6).

#### 3.2.7 SPT positive: Any allergy validation

An ‘any allergy’ algorithm‐outcome was assigned to 288/396 (72.7%) SPT‐positive participants (Table 5) with 87.5% (95% CI, 83.1–91.1) accuracy (Table 6). The algorithm showed very high sensitivity (90.2% [95% CI, 86.0–94.1]) and PPV (93.5% [95% CI, 88.4–96.4]), moderate specificity (78.1% [95% CI, 66.0–87.5]) and NPV (69.4% [95% CI, 57.5–79.8]) (Table 6), and good AUC (0.84 [95% CI, 0.79–0.90]) (Table 6).

### 3.3 Sensitivity analysis

A sensitivity analysis of SPT‐positive infants including definite allergy outcomes (excluding probable allergy) (Table S7) was performed. Algorithm classification of ‘any allergy’ was largely consistent with the primary analysis; however, due to low numbers, estimates were wider for each allergen.

## 4 DISCUSSION

In this paper we present the first validated research algorithm for classifying IgE‐mediated food allergies in infants from clinical trial and observational research cohorts. Validated using 740 OFCs undertaken in 587 research participants, algorithm‐derived outcomes demonstrated consistently high agreement with OFC outcomes across peanut, cashew, egg, and cow's milk (81%–97%). Minimal expert review and input were required, as the algorithm contained sufficient options to classify 92% of infants based on readily accessible and routinely collected data.

Our algorithm performance is particularly encouraging when compared to the only other research algorithm validated for younger children.^25^ Specifically, Kelleher and colleagues^10^ developed an algorithm to diagnose IgE‐mediated food allergy in 2‐3‐year‐olds, eligible for, but not undergoing, milk, egg or peanut OFC.^25^ The pediatric algorithm, validated for the 2‐year‐old Barrier Enhancement for Eczema Prevention (BEEP)^26^ and 3‐year‐old Enquiring About Tolerance (EAT) study participants,^27^ focused on identifying ‘any allergy’ rather than specific allergies; likely due to the smaller participant numbers in their validation cohorts (*n* = 124) compared to ours (*n* = 587). For algorithm validation, a BEEP expert panel blinded to OFC‐outcomes used the algorithm to guide decision making and determine allergy outcomes for 124 participants: incorporating SPT outcomes (with a single ‘highly sensitized’ > = 7 mm threshold for all allergens), and parent‐reported participant ingestion and reaction data. In contrast, we incorporated allergen specific SPT thresholds^14^, ^15^, ^16^ in our algorithm, and our comprehensive approach translated to minimal need for expert panel review in only 8% of cases. While the Kelleher et al.^10^ algorithm reported similar performance in terms of AUC and sensitivity, their estimates were imprecise due to missing SPT and clinical data. As a result, 55% of algorithm outcomes were inconclusive in the BEEP 2‐year‐old cohort. Comparatively, the large cohort of infants included in our algorithm validation, with conclusive outcomes (73%) and minimal missing data, helped to strengthen the precision of our analysis.

Although we had limited missing data, many infants had caregiver‐reported allergen ingestion that fell below our threshold for tolerance. Few participants in our study had been introduced to cashew by age 1 year, were ingesting peanut in sufficient quantity and frequency, or were ingesting egg in a form (soft‐ or non‐cooked) (data not shown), that met algorithm‐defined thresholds for tolerance. This translated to 27% of SPT‐positive infants, allocated an inconclusive ‘any allergy’ outcome. We had anticipated that a proportion of inconclusive algorithm outcomes would arise from inadequate allergen ingestion, as many parents remain hesitant to introduce allergens by 12‐months, despite recommendations.^28^ However, given the recent trends towards earlier allergen introduction^18^, ^29^ following updates to Australian feeding guidelines (2016),^30^ we anticipate ongoing improvements to rates of conclusive algorithm outcomes, with time. Despite this, it is important to note that the inconclusive rate remains an inherent feature of the algorithm and may therefore affect its utility. There are several strengths to this study. We included a large number of participants and OFCs across a range of SPT wheal sizes and allergens. The algorithm demonstrated meaningful utility in infants with moderately size SPT wheals (i.e. > = 2 mm to <95% PPV), determining an outcome for nearly half of infants in this range. The inclusion of three population‐based studies with uniform approaches to clinical allergy diagnosis, but diverse sampling frameworks, supports the generalisability of our algorithm across varied research settings. Further, in emphasizing clinical history (with well‐defined caregiver‐reported criteria) alongside SPT, our algorithm offers immediacy of results. It is a viable option for research studies anticipating low OFC participation, or resource‐limited settings, seeking to minimize logistical and economic burden, for participants, clinicians and research programs. Finally, although the algorithm misclassified 32 of the 276 positive OFCs (peanut, cashew, egg white or cow's milk) as tolerant, none were associated with severe reactions, supporting the algorithm's safety profile. Collectively, these strengths support the Vitality algorithm as an objective, resource‐efficient tool that minimizes need for expert clinician input, making it well suited for large‐scale studies.

Our infant algorithm has some limitations. It is validated only for the four foods which represent the highest local prevalence (Melbourne, Australia, prevalence: 8.2% egg^1^; 3.1% peanut^13^; 1.3% cashew^28^; 1.3% cow's milk^31^) and greatest research‐clinic demand (80% of the EarlyNuts and Vitality study OFCs performed). Due to the limited number of OFCs performed for other allergens, additional allergen validation was not feasible. Consequently, future research should include validating the algorithm in other common food allergens, such as sesame.

Additionally, as caregiver‐reported tolerance and reaction histories are susceptible to recall bias, this may contribute to some misclassification.^32^, ^33^ Specifically, inaccurate reporting of allergen ingestion or reaction symptoms could result in incorrect classification of participant tolerance or allergy. In this study, most infants misclassified as algorithm‐tolerant typically presented with convincing caregiver‐reported ingestion histories and low‐to‐moderate SPT sensitisation (data not shown). Notably, while our ingestion thresholds for caregiver‐reported tolerance surpass the cumulative ED50.0,^34^ they do not meet the cumulative dose requirements for OFC tolerance criteria.

The transient nature of food allergy in early childhood,^17^ may also contribute to algorithm misclassification. Most false‐positive outcomes for peanut and egg arose from convincing caregiver report of prior allergic reaction or independent doctor diagnosis of allergy, consistent with the clinical observations reported by others.^35^, ^36^ Additionally, we utilized history of soft‐cooked egg ingestion to estimate raw egg OFC tolerance, as caregiver‐reported raw egg ingestion is uncommon in the infant diet,^18^ and was poorly captured in our study. Despite this difference in egg preparations and as the literature suggests,^37^ soft‐cooked egg was moderately suited for predicting raw egg OFC tolerance in most low‐to‐moderately egg sensitized infants.

For these reasons, this algorithm is not a replacement for the gold‐standard OFC. However, quantifying its performance against gold‐standard OFC enhances the rigor of the outcomes derived. This algorithm has the potential to contribute to other allergy studies^3^ or contexts limited to SPT and clinical history by assigning outcomes, with reasonable accuracy, for up to 73% of participants who might otherwise have missing study outcome data. To establish broader relevance, it will be important to validate the algorithm in external populations beyond Australia, where allergy prevalence rates^38^, ^39^ and early allergen feeding patterns vary.^40^, ^41^

In conclusion, our algorithm offers a useful framework for classifying infant food allergy in settings where OFCs are not performed. To the best of our knowledge, we offer the first published, validated food allergy research algorithm specifically for infants, demonstrating strong performance for classifying peanut, cashew, egg, and cow's milk allergy.

## AUTHOR CONTRIBUTIONS

**Alexsandria Odoi:** Conceptualization; investigation; writing – original draft; methodology; validation; visualization; writing – review and editing; formal analysis; project administration; data curation. **Jennifer J. Koplin:** Conceptualization; investigation; funding acquisition; methodology; validation; writing – review and editing; project administration; data curation; supervision. **Victoria X. Soriano:** Investigation; writing – review and editing; data curation. **Kayla M. Parker:** Writing – review and editing; data curation. **Katrina J. Allen:** Funding acquisition; writing – review and editing; project administration. **Shyamali C. Dharmage:** Funding acquisition; writing – review and editing. **Katherine J. Lee:** Writing – review and editing. **Jana K. Eckert:** Writing – review and editing; project administration; data curation. **Audrey M. Walsh:** Writing – review and editing; project administration; data curation. **Angela Young:** Writing – review and editing; project administration. **Timothy W. Brettig:** Writing – review and editing; formal analysis. **Rachel L. Peters:** Conceptualization; methodology; validation; writing – review and editing; project administration; data curation; supervision. **Kirsten P. Perrett:** Conceptualization; methodology; validation; writing – review and editing; formal analysis; project administration; data curation; supervision; investigation; funding acquisition.

## FUNDING INFORMATION

The Vitality study was funded by the National Health and Medical Research Council (NHMRC) Australia (1146913); the National Institute of Health (NIH) Immune Tolerance Network (5UM1AI109565‐05); the DHB foundation; the Rotary Club of Camberwell; the Isabel and John Gilberston Charitable Trust; the Ilhan Food Allergy Foundation; the Kimberly Foundation Australia; the Constantinou Foundation; and other philanthropic donations. The EarlyNuts study was supported by funding from the NHMRC Australia (1146769) and the Murdoch Children's Research Institute. The HealthNuts study was funded by the NHMRC Australia (491233), the Ilhan Food Allergy Foundation, AnaphylaxiStop, and the Sylvia and Charles Viertel Charitable Foundation. Research at MCRI is supported by the Victorian Government's Operational Infrastructure Support Program. AO is supported by a Research Training Program PhD scholarship provided by the Australian Commonwealth Government and the University of Melbourne, and a PhD Top Up Scholarship provided by the Murdoch Children's Research Institute. JJK is supported by an NHMRC fellowship, 1158699. KPP is supported by an NHMRC fellowship, 2008911 and a Melbourne Children's Clinician–Scientist Fellowship. Audrey Walsh is supported by an Australian Government Research Training Program Scholarship provided by the Australian Commonwealth Government and the University of Melbourne, and a PhD scholarship from the Australian Government funded National Allergy Centre of Excellence (NACE), hosted by the Murdoch Children's Research Institute (MCRI), and their work was supported by the Victorian Government's Operational Infrastructure Support Program. KJL is supported by an NHMRC investigator grant, 2017498.

## CONFLICT OF INTEREST STATEMENT

KPP's institution has received research grants from Aravax, DBV Technologies, Novartis, and Siolta and consultant fees from Aravax, Novartis, and RAPT Therapeutics outside the submitted work. KPP is Chair of the Scientific Advisory Board of AllergyPal and a non‐executive director of OmnisOva. RLP's institution has received research grants from NHMRC, Stallergenes Greer Foundation, the National Peanut Board, and Allergy & immunology Foundation of Australia, paid to their institution outside of the submitted work. RLP has received speaker fees from Stallergenes Greer. There are no conflicts of interest declared by the other authors.

## Supporting information


Table S1.

## Data Availability

The data that support the findings of this study are available on request from the corresponding author. The data are not publicly available due to privacy or ethical restrictions.
