# Supplementary material for: The vitality infant algorithm: A validated tool to classify food allergy outcomes in research studies
Source: Pediatr Allergy Immunol. 2026 Oct 23;37(10):e70528. doi: 10.1111/pai.70528 (PMC13691615; doi:10.1111/pai.70528)
Supplement: Supplementary file 1 [file PAI-37-e70528-s001.docx]

Supplementary Material

Table of Contents

[Section 1: Algorithm inputs - data thresholds	2](#_Toc226891760)

[Allergy Assessments	2](#_Toc226891761)

[Caregiver (parent) reported allergic reaction	3](#_Toc226891762)

[Section 2: Algorithm Outputs (outcomes)	4](#_Toc226891763)

[Section 3: Oral food challenge dosing protocol	5](#_Toc226891764)

[Section 4: Alternative algorithm – sIgE focus	6](#_Toc226891765)

[Section 5: EarlyNuts and Vitality participant questionnaires	8](#_Toc226891766)

[Section 6: Algorithm performance by SPT threshold	10](#_Toc226891767)

[Section 7: Expert panel review	12](#_Toc226891768)

[Section 8: Algorithm validation sensitivity analysis (definite allergic)	14](#_Toc226891769)

[Supplementary material references	15](#_Toc226891770)

# Section 1: Algorithm inputs - data thresholds

## Allergy Assessments

### Vitality Skin prick test

Table S1 Algorithm SPT thresholds: sensitisation category by wheal size (mm)

| **Allergen** | **Negative** | **Positive** | | |
| --- | --- | --- | --- | --- |
|  |  | **Low** | **Moderately sensitised** | **Highly**  **Sensitised**  **(≥ 95 % PPV)** |
| **Peanut and Cashew** | 0 | 1 | 2 - 7 | ≥8 ^(1, 2)^ |
| **Egg** | 0 | 1 | 2 - 3 | ≥4 ^(2, 3)^ |
| **Cow’s milk** | 0 | 1 | 2 - 5 | ≥6 ^(3)^ |

Skin prick test thresholds have been based on published 95% positive predictive values (PPV) (1-3). mm: millimetres; OFC: oral food challenge.

Table S2 Comparison of EarlyNuts and Vitality participant self-reported questionnaire frequency data for 12-month-old infants.

| **Allergen** | **Frequency of Ingestion** | | **Minimum threshold met?**  **(for frequency)** |
| --- | --- | --- | --- |
|  | **EarlyNuts** | **Vitality** |  |
| All allergens | Once | Once | If selected at 2 or more time points* |
|  | 2-4 times | 2-4 times | Yes |
|  | More than 4 times  1 per week  2 per week  3 or more per week | 5 or more times | Yes |

*Vitality ingestion and reaction questionnaires were repeated when infants were aged 3, 6, 9 and 12 months old, so the selection of the option of “once” for ingestion history on ≥2 occasions, met the minimum frequency threshold.

## Caregiver (parent) reported allergic reaction

### Clearly defined allergic reaction

Consistent with previously published criteria (4), caregiver-reported allergic reaction met criteria for IgE-mediated allergic reaction if caregivers reported their infant experienced urticaria; perioral or periorbital angioedema or; vomiting, within 1 hour of allergen ingestion.

### Recent reaction

IgE-mediated allergic reactions occurring within 1 month of the SPT for egg, or within 2 months for cashew or peanut. A ‘non-recent’ reaction is one occurring outside these timeframes.

### Clearly defined case of anaphylaxis (ever)

In line with the ASCIA definition (5) of anaphylaxis, caregiver-report of their infant ever experiencing a food allergic reaction with symptoms of circulatory or respiratory compromise, following ingestion of the allergen of interest.

### Tolerance following a clear allergic reaction:

(Not shown in the algorithm illustration in figure 1) Where there is caregiver-report of allergen tolerance following a prior IgE-mediated allergic reaction or anaphylaxis, expert panel review (EPR) is required to determine if true tolerance has been demonstrated based on the quantity, frequency, and form that the allergen was consumed and tolerated in, prior to confirming algorithm tolerance.

# Section 2: Algorithm Outputs (outcomes)

Table S3 Conditions for the 5 algorithm outcomes and the expert panel review, as defined by our algorithm development panel.

| **Outcome** |  | **Assessment** |  |  | **Caregiver reported history** |
| --- | --- | --- | --- | --- | --- |
| (1) Definite Tolerant |  | Negative SPT (0mm) alone; or |  |  |  |
|  |  | Negative OFC (following positive SPT^a^) |  |  |  |
| (2) Probable Tolerant |  | Low^b^ to Moderate^c^ SPT | **AND** |  | Parent reported tolerance; or |
|  |  |  |  |  | Parent reported tolerance alone; or |
|  |  | Low^b^ SPT | **AND** |  | No history, or parent reported history that does not meet thresholds for tolerance or allergic reaction |
| (3) Definite Food Allergy |  | Positive OFC result alone; or |  |  | Anaphylaxis (ever) alone; or |
|  |  | Moderate^c^ to High^d^ SPT | **AND** |  | Anaphylaxis (ever); or |
|  |  | Low^b^, Moderate^c^, or High^d^ SPT | **AND** |  | Recent^e^ allergic reaction; or |
|  |  | High^d^ SPT | **AND** |  | Non-recent allergic reaction |
| **(4) Probable Food Allergy** |  | High^d^ SPT alone; or |  |  | Recent^e^ allergic reaction alone; or |
|  |  | Moderate^c^ SPT | **AND** |  | Non-recent^e^ allergic reaction |
| (5) Inconclusive Food Allergy |  | No SPT outcome, *or* a Moderate^c^ SPT | **AND** |  | No history, or parent reported history that does not meet thresholds for tolerance or allergic reaction |
| **(i) Expert Panel Review^f^** |  | Low^b^ SPT | **AND** |  | Anaphylaxis (ever); or |
|  |  | Low^b^ SPT | **AND** |  | Non-recent^e^ allergic reaction; or |
|  |  | High^d^ SPT | **AND** |  | Parent reported tolerance; or |
|  |  | Inconclusive OFC result; or |  |  | Non-recent^e^ allergic reaction alone; or |
|  |  |  |  |  | Parent reported tolerance following reaction |

^a^ Positive skin prick test (SPT) ≥ 1 mm.

^b^ Low SPT: Wheal 1mm.

^c^ Moderate SPT: Peanut and cashew wheal 1-7mm; egg 2-3mm; cow’s milk 2-5mm.

^d^ High SPT: Peanut and cashew wheal ≥ 8 mm; egg ≥ 4 mm; cow’s milk ≥ 6 mm.

^e^ Recent reaction: Egg and cow’s milk within 1 month of SPT; peanut and cashew within 2 months of SPT. Non-recent reactions are those that occur outside these defined periods.

^f^ Expert panel review: Team (≥2) of study and study-independent allergists, review participants with incongruous outcomes and data such as those detailed above. The panel can seek and review further data if available, such as study sIgE (*if* caregiver provided consent for blood sample collection), external (non-study) allergist results or reports, or additional correspondence between the study team and the participant caregivers. Following review of the participant data and results, the panel assign the participant to one of the 5 algorithm outcomes.

# Section 3: Oral food challenge dosing protocol

| **Allergen** |  | **Raw Egg White** | |  | **Peanut** | |  | **Tree Nut** | |  | **Cow’s milk** | |
| --- | --- | --- | --- | --- | --- | --- | --- | --- | --- | --- | --- | --- |
| **Dose interval** |  | 15 minutes | |  | 20 minutes | |  | 20 minutes | |  | 15 minutes | |
| **Product** |  | Pasteurised egg  (PUREGG® brand, Australia) ^c^ | |  | Smooth peanut butter paste (Bega® brand, Australia) ^d^ | |  | Crushed nut or nut meal  (e.g.: cashew, almond) | |  | Full cream dairy milk | |
| **Dose** |  | Quantity | Protein |  | Quantity | Protein |  | Quantity | Protein |  | Quantity | Protein |
| **1** |  | 0.05 ml^a^ | 2.8 mg |  | 1/16 tsp^a^ | 75 mg |  | Crumb  < 1/16 tsp | < 75 mg |  | 0.05 ml^a^ | 17 mg |
| **2** |  | 0.5 ml | 28 mg |  | 1/16 tsp^a^  (optional) | 75 mg |  | 1/16 tsp | 75 mg |  | 1.0 ml | 34 mg |
| **3** |  | 1.0 ml | 57 mg |  | 1/8 tsp | 150 mg |  | 1/8 tsp | 150 mg |  | 5.0 ml | 170 mg |
| **4** |  | 2.0 ml | 114 mg |  | 1/4 tsp | 300 mg |  | 1/4 tsp | 300 mg |  | 15.0 ml | 510 mg |
| **5** |  | 5.0 ml | 284 mg |  | 1/2 tsp | 600 mg |  | 1/2 tsp | 600 mg |  | 40.0 ml | 1360 mg |
| **6** |  | 10.0 ml | 568 mg |  | 1 tsp | 1200 mg* |  | 1 tsp | 1200 mg* |  | 100 ml | 3400 mg |
| **7** |  | 12.0 ml | 682 mg |  | - | - |  | - | - |  | - | - |
| **Total**  **(cumulative)dose** |  | **30.55 ml** | **1735 mg** |  | **9.7 – 10.0 g** | **2325 –2400 mg** |  | **~2 tsp** | **2400 mg** |  | **161.05 ml** | **5491 mg** |
| **Total dose equivalence** |  | 1 large egg  (60 g egg) | |  | 1.9 - 2 teaspoon  peanut butter | |  | ~2 teaspoon of  nut meal or crushed nut | |  | ~2/3 cup  of milk | |
| **Home continuation**  **Day 2 to 7^e^** |  | 1 whole egg white daily  30 ml | |  | 1 tsp daily | |  | 1 tsp daily | |  | Day 2: 100 ml  Day 3: 200 ml  Day 4-7: Equivalent of 3 serves of cow’s milk a day^b^ | |

Table S4 OFC protocol for the Vitality trial, EarlyNuts and HealthNuts studies

*Peanut and tree nut OFC top doses of 1200 mg are considered age appropriate and in line with current standard practice (Australia) for children under 2-years of age.

^a^ Drop (egg or milk) or smear (peanut butter paste) inside lip, do not touch outside lip.

^b^ Age dependent and considering current breastfeeding.

^c^ After the initial dose of a drop, mix in with age-appropriate foods.

^d^ Use a level metric spoon to quantitate each dose of peanut paste.

^e^ In the case of a negative OFC (day 1).

**g:** grams**; ml:** millilitres; **tsp**: tsp.

Eliciting dose 50 (ED50.0) discrete/cumulative (2): peanut 165 mg/236 mg; cashew 139 mg/232 mg; egg 94.5 mg/134 mg; milk 125 mg/192 mg

# Section 4: Alternative algorithm – sIgE focus

Figure S1 Hypothetical alternative algorithm for classifying allergy using sIgE to measure sensitisation (not validated).

^a^ Parent reported tolerance: Two or more occasions where the following are ingested; 2 or more bites of peanut butter or foods containing cashew, or non-cooked egg, or dairy containing foods (e.g. yogurt); or 5 or more peanuts or cashew nuts; or 1/2 a soft cooked egg; or 1-sip of cow’s milk. ^b^ Parent reported allergic reaction: Caregiver-report of symptoms consistent with sIgE allergic reaction of urticaria; perioral or periorbital angioedema or; vomiting, within 1 hour of allergen ingestion. ^c^ Non-recent reaction: Reaction occurring more than 1 month (Egg and Cow's Milk) or more 2 months (all other allergens) prior to sIgE. ^d^ Recent reaction: Occurring within 1 month (Egg and Cow's Milk) or 2 months (all other allergens) of sIgE^e^ Anaphylaxis: Defined as caregivers reporting symptoms consistent with the Australasian Society of Clinical Immunology (ASCIA) definition of anaphylaxis including circulatory or respiratory compromise. *Specific conditions apply for a report of anaphylaxis. Infants with a report of anaphylaxis and sIgE of 0.1 to < 0.35 kUL. require expert panel review (not shown in figure). Infants with a report of anaphylaxis and an sIgE of ≥ 0.35 kUL**,** receive an algorithm classification of definite allergic (as per figure). ^#^95% Positive predictive values (PPV) that are specific to the allergen, age and local setting of patients should be inserted here, by the algorithm user. ^ No history of ingestion or tolerance, or history did not meet the algorithm-specified threshold*. mm Millimetres; OFC: Oral food challenge; kUL: Kilounits per LitreImage created in Lucid (lucid.co)*

# Section 5: EarlyNuts and Vitality participant questionnaires

Examples study questionnaires completed by caregivers from the EarlyNuts and Vitality study.

Figure S2 EarlyNuts parent questionnaire

Figure S3 Sub-section of questions from the Vitality study 12-month-old participant questionnaire for food ingestion and allergic reaction history.

*Nutella^:^ Commercial product made with hazelnuts.

^If the parent/guardian selects peanut butter in section 2, peanut butter related questions appear in section 4. When other foods are selected in section 2, above questions are repeated, specific to that other food (not shown here).

#If parent/guardian selects peanut butter in section 6, questions relating to peanut butter appear in section 7-14. When other foods are selected in section 6, section 7-14 questions are repeated, specific to that food (not shown here).

# Section 6: Algorithm outcomes by SPT threshold

Table S5: Comparison of algorithm and OFC outcomes, across different skin prick test sensitisation thresholds (low, moderate and high) and allergens.

|  |  |  | **ORAL FOOD CHALLENGE OUTCOME (n=549)** | | | | | | **ALGORITHM PERFORMANCE SUMMARY** **n/N (%)** | |
| --- | --- | --- | --- | --- | --- | --- | --- | --- | --- | --- |
|  |  |  | **POSITIVE (n=365)** | | | **NEGATIVE (n=184)** | | | Conclusive algorithm outcomes (allergic or tolerant) | Agreement between conclusive algorithm outcomes & OFC |
| **ALLERGEN  (n OFCs)** | **SPT THRESHOLD**  **(n)** | **ALGORITHM OUTCOME** | Ingestion  History (n) | Reaction  History (n) | No History*  (n) | Ingestion  History (n) | Reaction  History (n) | No History*  (n) |  |  |
| **PEANUT  (n= 144)** | **Low: 1 mm (n=11)** | ALLERGIC | - | 1 | - | - | - | - | 11/11 (100%) | 10/11 (90.9%) |
|  |  | TOLERANT | - | - | 1 | 5 | - | 4 |  |  |
|  |  | INCONCLUSIVE | - | - | - | - | - | - |  |  |
|  | **Moderate:**  **2-7 mm**  **(n=114)** | ALLERGIC | - | 27 | 2 | - | 10 | - | 63/114 (55.3%) | 48/63 (76.2%) |
|  |  | TOLERANT | 5 | - | - | 18 | 1 | - |  |  |
|  |  | INCONCLUSIVE | - | - | 25 | - | - | 26 |  |  |
|  | **High: >=8 mm (n=19)** | ALLERGIC | - | 8 | 9 | - | - | 1 | 19/19 (100%) | 17/19 (89.5%) |
|  |  | TOLERANT | 1 | - | - | - | - | - |  |  |
|  |  | INCONCLUSIVE | - | - | - | - | - | - |  |  |
| **CASHEW  (n= 108)** | **Low: 1 mm (n=11)** | ALLERGIC | - | - | - | - | - | - | 11/11 (100%) | 11/11 (100%) |
|  |  | TOLERANT | - | - | - | 1 | - | 10 |  |  |
|  |  | INCONCLUSIVE | - | - | - | - | - | - |  |  |
|  | **Moderate:**  **2-7 mm**  **(n=84)** | ALLERGIC | - | 5 | - | - | - | - | 17/84 (20.2%) | 14/17 (82.4%) |
|  |  | TOLERANT | 3 | - | - | 9 | - | - |  |  |
|  |  | INCONCLUSIVE | - | - | 24 | - | - | 43 |  |  |
|  | **High: >=8 mm (n=13)** | ALLERGIC | 1 | - | 10 | - | - | 2 | 13/13 (100%) | 11/13 (84.6%) |
|  |  | TOLERANT | - | - | - | - | - | - |  |  |
|  |  | INCONCLUSIVE | - | - | - | - | - | - |  |  |
| **EGG  (n= 258)** | **Low: 1 mm (n=15)** | ALLERGIC | - | - | - | - | - | - | 14/15 (93.3%) | 8/14 (57.1%) |
|  |  | TOLERANT | 2 | 1 | 3 | 5 | - | 3 |  |  |
|  |  | INCONCLUSIVE | - | - | - | - | 1 | - |  |  |
|  | **Moderate:**  **2-3 mm**  **(n=99)** | ALLERGIC | - | 20 | 1 | - | 1 | - | 51/99 (51.5%) | 38/51 (74.5%) |
|  |  | TOLERANT | 11 | 1 | - | 15 | 2 | - |  |  |
|  |  | INCONCLUSIVE | - | - | 36 | - | - | 12 |  |  |
|  | **High: >=4 mm (n=144)** | ALLERGIC | 16 | 45 | 75 | 1 | 1 | 1 | 144/144 (100%) | 138/144 (95.8%) |
|  |  | TOLERANT | 3 | - | - | 1 | 1 | - |  |  |
|  |  | INCONCLUSIVE | - | - | - | - | - | - |  |  |
| **COWS MILK  (n= 39)** | **Low: 1 mm (n=2)** | ALLERGIC | - | - | - | - | - | - | 2/2 (100%) | 2/2 (100%) |
|  |  | TOLERANT | - | - | - | 1 | - | 1 |  |  |
|  |  | INCONCLUSIVE | - | - | - | - | - | - |  |  |
|  | **Moderate:**  **2-6 mm**  **(n=23)** | ALLERGIC | - | 12 | - | - | - | - | 19/23 (82.6%) | 19/19 (100%) |
|  |  | TOLERANT | - | - | - | 7 | - | - |  |  |
|  |  | INCONCLUSIVE | - | - | 4 | - | - | - |  |  |
|  | **High: >=6 mm (n=14)** | ALLERGIC | - | 8 | 4 | - | - | - | 14/14 (100%) | 13/14 (92.9%) |
|  |  | TOLERANT | 1 | - | - | - | 1 | - |  |  |
|  |  | INCONCLUSIVE | - | - | - | - | - | - |  |  |
| **ALL OFCs  (n= 549)** | **Low: 1 mm (n=39)** | ALLERGIC | - | 1 | - | - | - | - | 38/39 (97.4%) | 31/38 (81.6%) |
|  |  | TOLERANT | 2 | 1 | 4 | 12 | - | 18 |  |  |
|  |  | INCONCLUSIVE | - | - | - | - | 1 | - |  |  |
|  | **Moderate:**  **2mm-<95%PPV (n=320)** | ALLERGIC | - | 64 | 3 | - | 11 | - | 150/320 (46.9%) | 119/150 (79.3%) |
|  |  | TOLERANT | 19 | 1 | - | 49 | 3 | - |  |  |
|  |  | INCONCLUSIVE | - | - | 89 | - | - | 81 |  |  |
|  | **High:**  **>=95% PPV (n=190)** | ALLERGIC | 17 | 61 | 97 | 1 | 1 | 4 | 190/190 (100%) | 179/190 (94.2%) |
|  |  | TOLERANT | 5 | 1 | - | 1 | 2 | - |  |  |
|  |  | INCONCLUSIVE | - | - | - | - | - | - |  |  |

*or ingestion/reaction threshold not met. Abbreviations: OFC, oral food challenge; SPT skin prick test

# Section 7: Expert panel review

The expert panel reviewed participants with cases of incongruous allergy assessment outcomes and parent reported data and included one Vitality study allergist (K.P.P), and one study-independent allergist (T.W.B). To assist their review, the panel (blinded to OFC outcomes) could seek further data where available, such as study sIgE, external (non-study) allergist results or reports, or additional correspondence between the study team and the participant caregivers. Following review of the participant data and results, the panel assigned the participant to one of the 5 algorithm outcomes of probable or definite allergic, probable or definite tolerant, or inconclusive. The panel reviewed 51 cases, classifying 46 as either tolerant or allergic, and 5 cases as inconclusive. These panel outcomes were included in the algorithm validation results (table 5 and 6).

Table S6 Summary of expert panel review (EPR) outcomes compared to OFC diagnosed allergy and tolerance. EPR outcomes of probable and definite allergic were combined into one outcome of algorithm allergic, and outcomes of probable and definite tolerant combined into algorithm tolerant.

| **Reason for Expert Panel Review** | **Allergen** | **Expert Panel decision** | **OFC Outcome (n)** | |  | **Total participants for EPR (n)** |  | **EPR conclusive^a^ outcome agreement with OFC**  **n/N (%)**  **(excluding inconclusive)** |
| --- | --- | --- | --- | --- | --- | --- | --- | --- |
|  |  |  | **Positive** | **Negative** |  |  |  |  |
| **Highly sensitised by SPT^b^ and caregiver reported history of allergen tolerance**  **(n=29)** | **Peanut** | Allergic | 0 | 0 |  | 0 |  | 0/1 (0) |
|  |  | Tolerant | 1 | 0 |  | 0 |  |  |
|  |  | Inconclusive | 0 | 0 |  | 0 |  |  |
|  |  | **Total** | **1** | **0** |  | **1** |  |  |
|  | **Egg** | Allergic | 16 | 0 |  | 16 |  | 19/22 (86.4) |
|  |  | Tolerant | 3 | 3 |  | 6 |  |  |
|  |  | Inconclusive | - | - |  | - |  |  |
|  |  | **Total** | **19** | **3** |  | **22** |  |  |
|  | **Cashew** | Allergic | 1 | 0 |  | 1 |  | 1/1 (100) |
|  |  | Tolerant | 0 | 0 |  | 0 |  |  |
|  |  | Inconclusive | 0 | 0 |  | 0 |  |  |
|  |  | **Total** | **1** | **0** |  | **1** |  |  |
|  | **Cow’s milk** | Allergic | 1 | 0 |  | 1 |  | 3/3 (100) |
|  |  | Tolerant | 1 | 1 |  | 2 |  |  |
|  |  | Inconclusive | - | - |  | - |  |  |
|  |  | **Total** | **2** | **1** |  | **3** |  |  |
| **1 mm SPT wheal and caregiver reported history of allergic reaction**  **(n=1)** | **Peanut** | Allergic | 1 | - |  | - |  | 1/1(100) |
|  |  | Tolerant | - | - |  | - |  |  |
|  |  | Inconclusive | - | - |  | - |  |  |
|  |  | **Total** | **1** | **-** |  | **1** |  |  |
|  | **Egg** | Allergic | 0 | 0 |  | 0 |  | 0/2 (0)  [+1 inconclusive outcome] |
|  |  | Tolerant | 2 | 0 |  | 2 |  |  |
|  |  | Inconclusive | 0 | 1 |  | 1 |  |  |
|  |  | **Total** | **2** | **1** |  | **3** |  |  |
|  | **Cashew** | Allergic | - | - |  | - |  | Nil |
|  |  | Tolerant | - | - |  | - |  |  |
|  |  | Inconclusive | - | - |  | - |  |  |
|  |  | **Total** | **-** | **-** |  | **-** |  |  |
|  | **Cow’s milk** | Allergic | 0 | 0 |  | 0 |  | 2/2 (100) |
|  |  | Tolerant | 0 | 2 |  | 2 |  |  |
|  |  | Inconclusive | 0 | 0 |  | 0 |  |  |
|  |  | **Total** | **0** | **0** |  | **2** |  |  |
| **Algorithm classified inconclusive, with a caregiver report of doctor diagnosis of food allergy**  **(n=17)** | **Peanut** | Allergic | 2 | 0 |  | 2 |  | 2/2 (100)  [+2 inconclusive outcomes] |
|  |  | Tolerant | 0 | 0 |  | 0 |  |  |
|  |  | Inconclusive | 1 | 1 |  | 2 |  |  |
|  |  | **Total** | **3** | **1** |  | **4** |  |  |
|  | **Egg** | Allergic | 2 | 0 |  | 2 |  | 2/2 (100) |
|  |  | Tolerant | 0 | 0 |  | 0 |  |  |
|  |  | Inconclusive | 0 | 0 |  | 0 |  |  |
|  |  | **Total** | **0** | **0** |  | **2** |  |  |
|  | **Cashew** | Allergic | - | - |  | - |  | Nil |
|  |  | Tolerant | - | - |  | - |  |  |
|  |  | Inconclusive | - | - |  | - |  |  |
|  |  | **Total** | **-** | **-** |  | **-** |  |  |
|  | **Cow’s milk** | Allergic | 0 | 0 |  | 0 |  | 1/1 (100)  [+1 inconclusive outcome] |
|  |  | Tolerant | 0 | 1 |  | 1 |  |  |
|  |  | Inconclusive | 1 | 0 |  | 1 |  |  |
|  |  | **Total** | **1** | **1** |  | **2** |  |  |
| **Caregiver-reported subsequent tolerance, following a initial caregiver reported allergic reaction**  **(n=17)** | **Peanut** | Allergic | 0 | 0 |  | 0 |  | 1/1 (100) |
|  |  | Tolerant | 0 | 1 |  | 1 |  |  |
|  |  | Inconclusive | 0 | 0 |  | 0 |  |  |
|  |  | **Total** | **0** | **1** |  | **1** |  |  |
|  | **Egg** | Allergic | 12 | 0 |  | 12 |  | 13/14 (92.9) |
|  |  | Tolerant | 1 | 1 |  | 2 |  |  |
|  |  | Inconclusive | 0 | 0 |  | 0 |  |  |
|  |  | **Total** | **13** | **1** |  | **14** |  |  |
|  | **Cashew** | Allergic | - | - |  | - |  | Nil |
|  |  | Tolerant | - | - |  | - |  |  |
|  |  | Inconclusive | - | - |  | - |  |  |
|  |  | **Total** | **-** | **-** |  | **-** |  |  |
|  | **Cow’s milk** | Allergic | - | - |  | - |  | Nil |
|  |  | Tolerant | - | - |  | - |  |  |
|  |  | Inconclusive | - | - |  | - |  |  |
|  |  | **Total** | **-** | **-** |  | **-** |  |  |
| **All reasons**  **(n=64)** | **All allergens** |  |  |  |  |  |  | 44/52 (84.6)  [+4 inconclusive outcomes] |

^a^Conclusive outcome: Panel determined outcome of either allergen allergic or tolerant. Panel determined inconclusive outcomes were excluded from agreement analysis but are shown in the table within square brackets. ^b^**Highly sensitised by SPT:** SPT greater than or equal to the 95% positive predictive value (PPV) : peanut (2) and cashew (1) ≥ 8 mm; egg (2)≥ 4 mm; cow’s milk (3) ≥ 6 mm. **EPR:** Expert panel review

# Section 8: Algorithm validation sensitivity analysis (definite allergic)

Table S7 *Algorithm validation sensitivity analysis, including definite allergy outcomes, in the EarlyNuts, Vitality and combined groups, for peanut, cashew, egg, cow’s milk and ‘any allergy’.*

| **Allergen** | **Study** | **Algorithm Outcome** | **OFC Outcome (n)** | | **Total conclusive algorithm outcomes (n)** |  | **Agreement**  **(95 CI)** | **Sensitivity**  **(95 CI)** | **Specificity**  **(95 CI)** | **PPV**  **(95 CI)** | **NPV**  **(95 CI)** | **AUC**  **(95 CI)** |
| --- | --- | --- | --- | --- | --- | --- | --- | --- | --- | --- | --- | --- |
|  |  |  | **Positive** | **Negative** |  |  |  |  |  |  |  |  |
| **Peanut** | **EarlyNuts** | Allergic | 5 | 0 | 5 |  | 81.3  (54.4 – 96.0) | 62.5  (24.5 - 91.5) | 100  (63.1 - 100) | 100  (47.8 - 100) | 72.7  (39.0 - 94.0) | 0.81  (0.63 – 0.99) |
|  |  | Tolerant | 3 | 8 | 11 |  |  |  |  |  |  |  |
|  |  | **Total** | **8** | **8** | **16** |  |  |  |  |  |  |  |
|  | **Vitality** | Allergic | 3 | 0 | 3 |  | 85.2  (66.3 – 95.8) | 42.9  (9.9 - 81.6) | 100  (82.4 - 100) | 100  (29.4 - 100) | 83.3 (62.6 - 95.3) | 0.71  (0.52 - 0.91) |
|  |  | Tolerant | 4 | 20 | 24 |  |  |  |  |  |  |  |
|  |  | **Total** | **7** | **20** | **27** |  |  |  |  |  |  |  |
|  | **Combined** | Allergic | 8 | 0 | 8 |  | 83.7  (69.0 – 93.2) | 53.3  (26.6 - 78.7) | 100  (87.7 - 100) | 100  (63.1 - 100) | 80.0 (63.1 - 91.6) | 0.77  (0.64 - 0.90) |
|  |  | Tolerant | 7 | 28 | 35 |  |  |  |  |  |  |  |
|  |  | **Total** | **15** | **28** | **43** |  |  |  |  |  |  |  |
| **Cashew** | **EarlyNuts** | Allergic | 0 | 0 | 0 |  | 100  (59.0 – 100) | * | 100  (2.5 – 100) | * | * | * |
|  |  | Tolerant | 0 | 7 | 7 |  |  |  |  |  |  |  |
|  |  | **Total** | **0** | **7** | **7** |  |  |  |  |  |  |  |
|  | **Vitality** | Allergic | 1 | 0 | 1 |  | 82.4  (56.6 – 96.2) | 25.0  (0.6 – 80.6) | 100  (75.3 – 100) | 100  (2.5 – 100) | 81.3  (54.4 – 96.0) | 0.63  (0.38 – 0.87) |
|  |  | Tolerant | 3 | 13 | 16 |  |  |  |  |  |  |  |
|  |  | **Total** | **4** | **13** | **17** |  |  |  |  |  |  |  |
|  | **Combined** | Allergic | 1 | 0 | 1 |  | 87.5  (67.6 – 97.3) | 25.0  (0.6 – 80.6) | 100  (83.2 – 100) | 100  (2.5 – 100) | 87.0  (66.4 – 97.2) | 0.63  (0.38 – 0.87) |
|  |  | Tolerant | 3 | 20 | 23 |  |  |  |  |  |  |  |
|  |  | **Total** | **4** | **20** | **24** |  |  |  |  |  |  |  |
| **Egg** | **EarlyNuts** | Allergic | 5 | 0 | 5 |  | 52.0  (31.3 – 72.2) | 29.4  (10.3 - 56.0) | 100  (63.1 - 100) | 100  (47.8 - 100) | 40.0  (19.1 - 64.0) | 0.65  (0.54 - 0.76) |
|  |  | Tolerant | 12 | 8 | 20 |  |  |  |  |  |  |  |
|  |  | **Total** | **17** | **8** | **25** |  |  |  |  |  |  |  |
|  | **Vitality** | Allergic | 31 | 1 | 32 |  | 83.3  (71.4 – 91.7) | 77.5  (61.6 - 89.2) | 95.0  (75.1 - 99.9) | 96.9  (83.8 - 99.9) | 67.9  (47.7 - 84.1) | 0.86  (0.78 - 0.94) |
|  |  | Tolerant | 9 | 19 | 26 |  |  |  |  |  |  |  |
|  |  | **Total** | **40** | **20** | **60** |  |  |  |  |  |  |  |
|  | **Combined** | Allergic | 36 | 1 | 37 |  | 74.1  (63.5 – 83.0) | 63.2  (49.3 – 75.6) | 96.4  (81.7 - 99.9) | 97.3  (85.8 - 99.9) | 56.3  (41.2 - 70.5) | 0.80  (0.73 – 0.87) |
|  |  | Tolerant | 21 | 27 | 48 |  |  |  |  |  |  |  |
|  |  | **Total** | **57** | **28** | **85** |  |  |  |  |  |  |  |
| **Cow’s**  **milk** | **EarlyNuts** | Allergic | 1 | 0 | 1 |  | 100  (54.1 – 100) | 100  (2.5 - 100) | 100  (47.8 – 100) | 100  (2.5 - 100) | 100  (47.8 - 100) | 1  (0 - 1) |
|  |  | Tolerant | 0 | 5 | 5 |  |  |  |  |  |  |  |
|  |  | **Total** | 1 | 5 | 6 |  |  |  |  |  |  |  |
|  | **Vitality** | Allergic | 7 | 0 | 7 |  | 92.3  (64.0 – 99.8) | 87.5  (47.4 - 99.7) | 100  (47.8 - 100) | 100  (59.0 - 100) | 83.3  (35.9 - 99.6) | 0.94  (0.82 - 1) |
|  |  | Tolerant | 1 | 5 | 6 |  |  |  |  |  |  |  |
|  |  | **Total** | 8 | 5 | 13 |  |  |  |  |  |  |  |
|  | **Combined** | Allergic | 8 | 0 | 8 |  | 94.7  (74.0 – 99.9) | 88.9  (51.8 - 99.7) | 100  (69.2 - 100) | 100  (63.1 - 100) | 90.9  (58.7 - 99.8) | 0.94  (0.84 - 1) |
|  |  | Tolerant | 1 | 10 | 11 |  |  |  |  |  |  |  |
|  |  | **Total** | 9 | 10 | 19 |  |  |  |  |  |  |  |
| **Any**  **Allergy^a^** | **EarlyNuts** | Allergic | 10 | 0 | 10 |  | 68.6  (50.7 – 83.1) | 47.6  (25.7 – 70.2) | 100  (76.8 - 100) | 100  (69.2 – 83.7) | 56.0 (34.9 – 75.6) | 0.74 (0.63 - 0.85) |
|  |  | Tolerant | 11 | 14 | 25 |  |  |  |  |  |  |  |
|  |  | **Total** | **21** | **14** | **35** |  |  |  |  |  |  |  |
|  | **Vitality** | Allergic | 41 | 0 | 41 |  | 87.5  (78.7 – 93.6) | 78.8 (65.3 - 88.9) | 100  (90.3 - 100) | 100  (91.4 - 100) | 76.6  (62.0 – 87.7) | 0.89  (0.84 - 0.95) |
|  |  | Tolerant | 11 | 36 | 47 |  |  |  |  |  |  |  |
|  |  | **Total** | **52** | **36** | **88** |  |  |  |  |  |  |  |
|  | **Combined** | Allergic | 51 | 0 | 51 |  | 82.1  (74.2 – 88.4) | 69.9  (58.0 - 80.1) | 100  (92.9 - 100) | 100  (93.0 - 100) | 69.4  (57.5 – 79.8) | 0.85  (0.80 - 0.90) |
|  |  | Tolerant | 22 | 50 | 72 |  |  |  |  |  |  |  |
|  |  | **Total** | **73** | **450** | **123** |  |  |  |  |  |  |  |

^a^ Any Allergy combines allergy outcome for participants with multiple oral food challenges (OFC): infants are deemed allergic if allergic to at least one food, and tolerant if tolerant to all foods. Calculations of accurate diagnosis, sensitivity, specificity, positive predictive values (PPV), negative predictive values (NPV), odds ratio (OR) and Area Under the Curve (AUC) include algorithm classifications of definite allergic and tolerant (excluding inconclusive and probable allergic). *Unable to calculate.

# Supplementary material references

1.	Ho MH, Heine RG, Wong W, Hill DJ. Diagnostic accuracy of skin prick testing in children with tree nut allergy. J Allergy Clin Immunol. 2006;117(6):1506-8.

2.	Peters RL, Allen KJ, Dharmage SC, Tang ML, Koplin JJ, Ponsonby AL, et al. Skin prick test responses and allergen-specific IgE levels as predictors of peanut, egg, and sesame allergy in infants. J Allergy Clin Immunol. 2013;132(4):874-80.

3.	Hill DJ, Heine RG, Hosking CS. The diagnostic value of skin prick testing in children with food allergy. Pediatr Allergy Immunol. 2004;15(5):435-41.

4.	Osborne NJ, Koplin JJ, Martin PE, Gurrin LC, Thiele L, Tang ML, et al. The HealthNuts population-based study of paediatric food allergy: validity, safety and acceptability. Clin Exp Allergy. 2010;40(10):1516-22.

5.	ASCIA. Anaphylaxis. Australasian Society of Clinical and Immunology. 2021;2022(08 May 2023).
